# Pilot Study on the Effect of Cannabidiol-Coated Fabric for Pillow Covers Improves the Sleep Quality of Shift Nurses

**DOI:** 10.3390/healthcare13060585

**Published:** 2025-03-07

**Authors:** Mashita Afzal, Chieh-Liang Huang, Shih-Hao Huang, Chia-Ing Li, Wen-Chun Liao, Juan-Cheng Yang, Wen-Lung Ma

**Affiliations:** 1 Graduate Institute of Biomedical Sciences, School of Medicine, China Medical University, Taichung 40403, Taiwan; mashitaafzal@gmail.com (M.A.); 006446@tool.caaumed.org.tw (C.-I.L.); 2 TsaoTun Psychiatric Center, Caotun Township, Nantou 54249, Taiwan; psyche.hcl@gmail.com; 3 SONG BEAM International Co., Ltd., Taichung 40650, Taiwan; louis@songbeam.com.tw; 4 Department of Medical Research, China Medical University Hospital, Taichung 40403, Taiwan; 5 School of Nursing, China Medical University, Taichung 40604, Taiwan; iwcl@mail.cmu.edu.tw; 6 Graduate Institute of Integrated Medicine, School of Chinese Medicine, China Medical University, Taichung 40403, Taiwan

**Keywords:** cannabidiol (CBD), pillowcase, non-rapid eye movement (NREM), sleep quality, nurses

## Abstract

**Background:** Sleep difficulty is common in the current society. Poor sleep has a significant influence on health, social interactions and even mortality; therefore, maintaining good sleep is of prime importance. Cannabidiol (CBD), a cannabis-derived compound, is known for its medical significance with many positive effects in humans, including decreasing anxiety and improving sleep for those with sleep disorders. **Objective:** However, whether CBD skin absorption results in similar effects is unknown. Therefore, examining CBD-coated fabric as a pillow cover to improve sleep quality in duty shift nurses is the purpose of this paper. **Methods:** This study recruited 55 duty shift nurses as participants to evaluate sleep patterns and quality using the Pittsburgh Sleep Quality Index (PSQI) and a consumer-grade tracker (Fitbit Charge 3). Data were collected over three phases: a one-week baseline period, a two-week intervention period using a CBD-coated pillow cover and a one-week follow-up period, referred to as the post-intervention phase, during which the use of CBD-coated pillow cover was continued. **Results:** Of the 55 participants, 10 were men (18.2%) and 45 were women (81.8%). At baseline, all participants exhibited poor sleep quality (PSQI ≥ 5). However, after three weeks of using CBD-coated pillow covers, subjective sleep quality significantly improved, with 7.3% of participants achieving PSQI scores <5. Additionally, slight changes in sleep patterns were observed, with increases in both light sleep and deep sleep durations. Light sleep duration increased from a baseline of 196.21 ± 65.28 to 206.57 ± 59.15 min two weeks after intervention (*p* = 0.337). Similarly, deep sleep duration showed a modest increase from 61.97 ± 21.01 min to 64.35 ± 22.19 min (*p* = 0.288). Furthermore, a significant reduction in anxiety levels was reported (*p* < 0.005). **Conclusions:** Using a CBD-coated pillow cover was found to enhance sleep duration in healthy individuals experiencing poor sleep. Consequently, for adults struggling with sleep difficulties, incorporating a CBD-coated pillow cover may serve as an effective aid in improving sleep quality.

## 1. Introduction

Poor sleep affects up to one-third of the global population and is considered a public health epidemic [1]. Various causes, such as strenuous workloads for professional achievement, may contribute to poor sleep, which has a high impact on health, family life and even mortality [2]. The maintenance of optimal sleep quality is an important issue, especially for individuals working rotating shifts or permanent night shifts, who experience transient or chronic sleep disturbance or even sleep deprivation [3]. Nurses working in rotating shifts or permanent night shifts have reported transient or chronic sleep disorders [4]. In night shift workers, sleep is usually initiated 1 h (SD = 30–60 min) after the termination of the shift [5,6]. Sleep is reduced by 2–4 h, with longer sleep latency and sleep fragmentation [7,8]. After a night shift, sleep loss mainly involves stage 2 and rapid eye movement (REM) sleep, whereas slow-wave sleep (SWS) stages 3 and 4 remain unaffected [9]. Day sleep after night work is short; hence, a late afternoon nap of >1 h before the subsequent night shift is added [10]. Night work is also characterized by increased subjective, behavioral and physiological sleepiness [11,12]. The effects are particularly severe in the early morning and often involve incidents of involuntary sleep. It usually takes two nights of sleep periods to recover from the last night shift back to the normal low sleepiness level. The increased sleep deprivation may be associated with an increased risk of accidents and errors and a feeling of fatigue [13,14]. In the morning shift, the sleep pattern before a shift appears to be even more disturbed compared to the night shift. EEG studies show that sleep duration is reduced by 2–4 h, with mainly stage 2 and REM being affected, whereas slow-wave sleep (SWS) stages 3 and 4 remain unaffected. Subjective complaints about morning shifts include difficulty in awakening, non-spontaneous awakening and a feeling of not being refreshed by sleep [7]. The ability to tolerate and adjust to shift work varies significantly between individuals, being influenced by factors such as chronotype (preferred time of activity), health status, sleep habits and social characteristics [14]. Shift system features, including the length of the shift, speed and direction of rotation and time of changeover, affect the adaptation to shift work. Some studies suggest that a schedule consisting of four consecutive night shifts, a clockwise rotation (morning–afternoon–night) and an end time around 07:00 may optimize performance and minimize sleep disruptions. Usually, 1–2 days are needed to adjust to a change in the shift, with a longer interval being better. Previous studies showed that employees preferred a 21-day schedule of fixed shifts, which resulted in better health, morale and performance and lower personnel turnover compared to the company average [15,16]. This type of fixed rotating shift over one month is adopted by most hospital shift systems. Good shift-work adaptors usually have better daytime sleep before a night shift, fewer social and family disruptions and greater alertness during the night shift than poor shift-work adaptors [17]. To enhance shift-work adaptation, countermeasures are still needed at work and in activity–rest schedules [14]. In addition to calmness before and during the resting time, which is necessary for better sleep-in shift nurses, it is crucial to address the challenges faced by nurses working in such conditions. This study aims to explore alternative approaches to mitigating the effects of sleep deprivation and improving recovery, which is of paramount importance in promoting the health and well-being of shift-working nurses.

Cannabidiol (CBD) is a biologically active compound found in the *Cannabis* plant. CBD has been known previously to reduce pain and alleviate a range of neurological conditions, including severe seizures, depression, anxiety and neuroprotection. In a crossover trial, CBD was found to extend sleep duration [18,19]. Several studies have highlighted that CBD may have dose-dependent side effects and potential addiction-related risks when used orally. However, controlled use of CBD appears to be challenging but generally well tolerated, with only a few patients reporting mild side effects, such as fatigue, mild sedation, increased inappropriate sexual behavior and dry eyes, which may be linked to dosage. A clinical trial (NCT02548559) demonstrated the efficacy of a 4-week treatment with CBD, showing favorable results with limited side effects [18,20,21]. Several studies suggest that CBD holds therapeutic potential for managing anxiety, insomnia and epilepsy. However, the optimal oral use of CBD remains a challenge and requires further investigation to better understand its safety and therapeutic potential; as an alternative approach to address this challenge, we examined the effects of a pillow cover made with CBD-coated fabric on sleep in nurses undertaking shift work. By offering an alternative to traditional CBD consumption methods, such as oral ingestion or vaping, the CBD-coated pillow may minimize potential side effects while still delivering therapeutic benefits. This innovative approach could provide a safer and more effective means of utilizing CBD to improve sleep quality, particularly for individuals with insomnia or those exposed to shift-work-related sleep disturbances.

## 2. Materials and Methods

### 2.1. Study Design and Procedure

**Figure 1:**
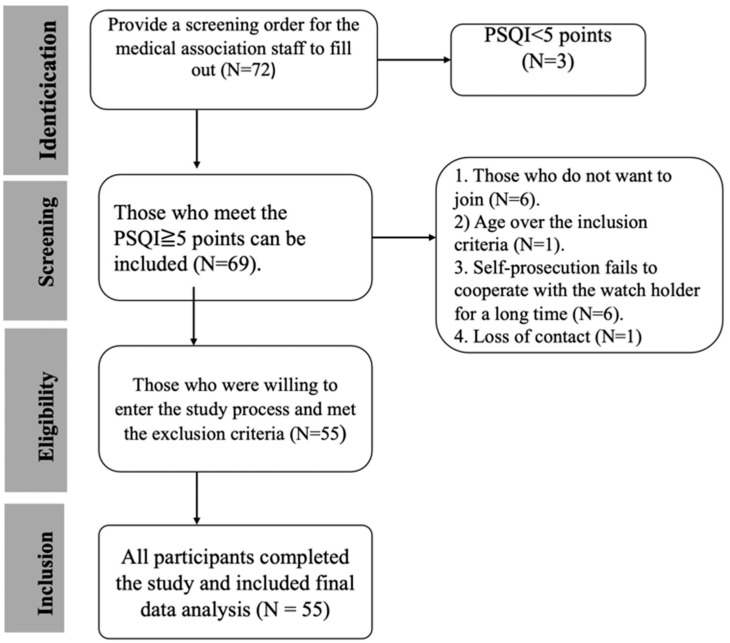
Flowchart showing the inclusion and exclusion criteria.

**Figure 2:**
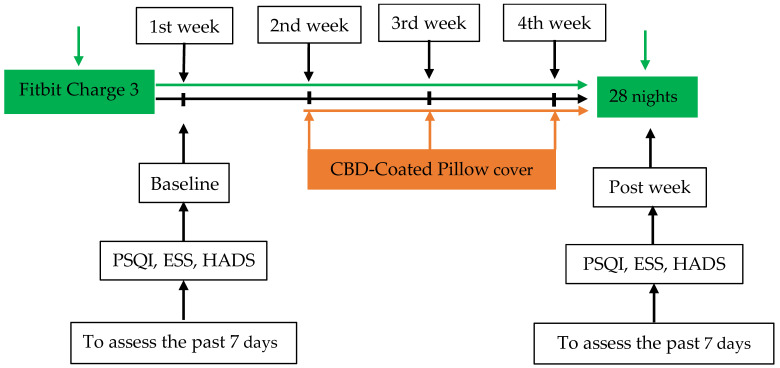
Flowchart illustrating the study design of CBD-coated pillow cover.

**Figure 3:**
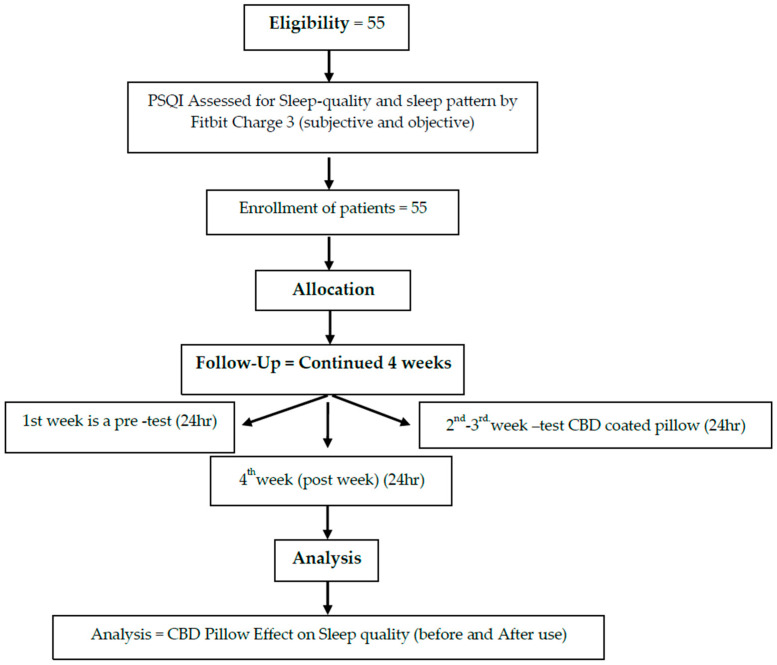
Flowchart showing the methodology of CBD-coated pillow intervention.

This pilot study recruited 55 members of medical staff from hospitals affiliated with the China Medical University (Figure 1). The participants were asked to wear a consumer tracker, a Fitbit active sleep watch (Fitbit Charge 3), on their wrists to record active sleep patterns and sleep quality during a baseline in the first week while using a pillow with a CBD-coated cover in the second week, during follow-up for one week and finally during a post-test at the end of the overall study (week 4). The study period was a total of four weeks. Participants maintained their habitual activity and sleep schedule. Sleep quality (both subjective and objective) was assessed using the Pittsburgh Sleep Quality Index (PSQI) and Fitbit Charge 3. In addition, the Epworth Sleepiness Scale (ESS) and Hospital Anxiety and Depression Scale (HADS) were applied to rate related changes, such as pre- or baseline test and post-intervention drowsiness and mood (Figure 2 and Figure 3).

### 2.2. Sample Size

Participants were recruited from the China Medical University Hospital, located in Taichung City. The sample of 55 subjects included 45 women (81.8%) and 10 men (18.2). Participants aged 21~49 with poor sleep quality (PSQI score >5) were recruited (Figure 1). Most participants were aged between 20~ and 40 years, with groups aged 21–30 years (49.1%) and 31–40 years (36.4%) comprising the majority. The work patterns of the subjects in the past three months were mainly fixed day shifts (58.2%), with 12 individuals (21.8%) working rotating shifts. During the second phase, participants used a pillow with a CBD-coated cover for approximately two weeks, continuing one week to the post-experiment period (Figure 2 and Figure 3).

### 2.3. Measures

Pre- and post-test sleep quality was measured using the PSQI. In addition, participants wore a Fitbit Charge 3 bracelet for 28 days to capture the total sleep time, light sleep per day, deep sleep, REM, wakefulness after sleep onset (WASO) and wake-up time and to calculate sleep efficiency and sleep distribution percentages (Figure 2). The total score of the PSQI is 0–21 points; the higher the score, the worse the sleep quality, with a cut-off of ≥5 points indicating poor sleep quality. Data on demographic parameters, including age, sex, education, marital status and work, the use of hypnotics and exercise habits, were also recorded. Sleep patterns and sleep quality were assessed.

#### 2.3.1. Sleep

Sleep patterns were measured using Fitbit Charge 3. Participants wore the tracker for four weeks continuously, except when taking a shower (Figure 2). Data on sleep patterns included the number of minutes and percentage of total sleep time, awake time, light sleep, deep sleep, REM sleep and WASO. Fitbit Charge 3 has been shown to be accurate in measuring sleep [22] and mobility [23].

#### 2.3.2. Sleep Quality

The PSQI was used to assess the habitual sleep of adults at baseline and after intervention over a two-week interval in the fourth week (Figure 2). It consists of nineteen self-rated questions that yield seven components: subjective sleep quality, sleep latency, sleep duration, habitual sleep efficiency, sleep disturbances, use of sleeping medication and daytime dysfunction. A global PSQI score is obtained by summing the scores for the components, with a range of 0–21. A higher score indicates worse sleep quality. A post hoc score of ≥5 is the cut-off point to discriminate between a “good” sleeper and a “poor” sleeper. The overall Cronbach’s alpha, the global PSQI for internal consistency and reliability, ranged from 0.77 to 0.83 [24,25]. The sensitivity and specificity of the PSQI was 80–89.6% and 86.5–86.6%, respectively [24,25,26].

### 2.4. Data Analysis

To assess the effectiveness of the CBD-coated pillow cover in sleep quality, inferential statistics were employed. First, the Shapiro–Wilk test was used to test the normality of each variable. Descriptive statistics were then calculated and presented as means ± standard deviations, medians, interquartile ranges, frequencies, percentages. Based on the distribution of the data, appropriate inferential statistics were selected. McNemar change tests were used for categorical data to compare sleep quality before and after pillow cover use. For normally distributed data, paired-sample t-tests and time series analysis were conducted. For non-normally distributed data, Wilcoxon signed-rank tests were utilized. Furthermore, to explore the factors associated with changes in sleep outcomes following the intervention, stepwise multiple regression analyses were conducted. All tests were two-sided, with a significance level of *p* < 0.05.

## 3. Results

The sample of 55 subjects included 45 (81.8%) women. The participants’ ages were mostly in the range between 20 and 40 years (85.5%). The work pattern of the participants in the previous three months was mainly fixed day shifts (58.2%), with 12 individuals (21.8%) working rotating shifts. Ten participants took hypnotics, and fifteen had an exercise habit (Table 1). The sleep quality of all participants was poor (PSQI ≥ 5), with a median score of 9.0 (interquartile range: 6.0, 12.0) at baseline (Table 2). The global PSQI decreased to 7.0 (6.0, 9.0) two weeks after intervention (Table 2). Four participants showed improvement in sleep (*p* < 0.1) (Table 3). They slept about 6 h a night, with 86.3 sleep efficiency and 48.1% of the sleep being light sleep and 15.8% deep sleep (Table 4). The total study period was 28 days, which consisted of pre-test in the first week, using a pillow with a CBD-coated cover in the second week, a follow-up for one week and finally a post-test at the end of the overall study (week 4). The sleep quality in four participants improved to 7.3% (Table 3). The Subjective Sleep Quality score remained consistent in terms of the median at 1.0 (1.0, 2.0) between baseline and post-intervention, as measured by PSQI. Changes in scores for the drowsiness and HADS subscales for anxiety and depression were analyzed using paired *t*-tests (Table 2). The CBD intervention resulted in statistically significant improvements across all measures (all *p* < 0.05).

### 3.1. Sleep Quality (Subjective)

The 55 participants had an average overall score of 9.0 for subjective sleep quality at baseline. After three weeks of intervention, the overall sleep quality score decreased to 7.0 (*p* < 0.001; Table 2); the sleep quality of 100% of the participants was poor (PSQI ≥ 5; Table 3). The total number of sleep hours (minutes) at baseline 362.29 ± 63.72 increased to 365.18 ± 63.71 two weeks after intervention, and participants had longer sleep latency mostly due to the following reasons: inability to fall asleep within minutes; getting up to use the toilet; not easy to fall asleep again after waking up in the middle of the night; and the influence of a hot or cold environment. Table 3 shows that three weeks after using the CBD-coated pillow cover, subjective sleep quality (PSQI < 5) improved in four participants (7.3%; *X*^2^ = 10.93, *p* = 0.091).

### 3.2. Sleep Quality (Objective)

Table 4 shows the CBD-coated pillow cover intervention from baseline (7 days) to 2 weeks after intervention with CBD-coated pillow cover and with one week of follow-up, for a total of 28 days. The results of statistical tests did not find a significant difference in sleep structure time but found a difference in sleep structure distribution. There was a slight increase in light sleep duration from baseline 196.21 ± 65.28 to 206.57 ± 59.15, *p* = 0.337 after the intervention (Table 4). Deep sleep time increased slightly from 61.97 min a night at baseline to 64.35 min a night during the intervention.

### 3.3. Mood and Lethargy

Table 2 shows the ESS scores for the total 21 days of the intervention using the CBD-coated pillow cover, from baseline (7 days) to two weeks of follow-up with CBD-coated pillow cover and one week after CBD intervention. The total scores for drowsiness and HADS (depression and anxiety) were compared using a paired *t*-test. Compared to improved sleep quality, the CBD intervention significantly reduced anxiety (*p* < 0.005). Reduced anxiety levels may contribute to improved sleep quality, as anxiety often interferes with the ability to fall asleep and maintain restful sleep. A reduction in depressive symptoms could also support better sleep quality, as depression often impacts sleep patterns negatively (e.g., difficulty initiating or maintaining sleep) [27], making the pillow an effective tool for improving overall mental and sleep health.

### 3.4. Factors Influencing Intervention Efficacy

The impact of the intervention seemed to be related to whether the participants had an exercise habit; if they had an exercise habit, the impact of the CBD intervention on their sleep was objectively less insensitive, but if they did not have an exercise habit, it showed a more obvious or negative effect on sleep quality. Of those without an exercise habit, the overall objective sleep efficiency trend improved in 20 (36.4%) individuals, and the trend worsened or did not change in 20 participants.

To elucidate the factors associated with post-intervention changes in sleep outcomes, stepwise multiple regression analyses were performed. The dependent variables included changes in the global Pittsburgh Sleep Quality Index (PSQI) score (ΔPSQI), Hospital Anxiety and Depression Scale (HADS), anxiety subscale score (ΔHADS-A), HADS depression subscale score (ΔHADS-D) and light sleep percentage (ΔLS%). The following potential predictors were incorporated into the models: sex, age, marital status, work schedule during the preceding year and three months, use of adjunctive sleep medications and exercise habits. Notably, exercise habits did not emerge as a significant predictor in these regression models.

We found significant predictors for ΔPSQI, ΔHADS-D and ΔLS%, while no significant predictors emerged for the change in anxiety score (ΔHADS-A). Males were associated with a significantly greater decrease in global PSQI score (ΔPSQI = 2.23, *p* = 0.037), indicating greater improvement in sleep quality compared to females. Males were associated with a significantly greater decrease in HADS depression subscale score (ΔHADS-D = 1.98, *p* = 0.044), indicating greater improvement in depression compared to females. Participants working fixed night shifts in the past 3 months had a significantly greater decrease in global PSQI scores compared to those working fixed day shifts (β = 2.98, *p* = 0.049). Being in the 31–40 age group was associated with a greater increase in light sleep percentage compared to the 21–30 age group (ΔLS% = −3.18, *p* =.044). Taking adjunct sleeping medications was associated with a decrease in light sleep percentage (ΔLS% = −3.55, *p* = 0.028).

## 4. Discussion

Sleep is a vital process, especially for shift workers such as nurses. CBD shows promise for improving sleep quality, potentially through its interaction with the skin’s endocannabinoid system (ECS), which facilitates absorption and localized therapeutic effects [28].

Daytime sleep after night work is typically short, prompting the addition of late afternoon naps before subsequent night shifts. [29]. Cannabidiol has been considered by many studies to be safe for short-term use. It has a neuroprotective role in Alzheimer’s and Parkinson’s diseases, anti-inflammatory and antioxidant properties, making it valuable for treating various diseases [30,31]. Therefore, it has the potential to be an important part of the treatment for insomnia patients. After three weeks of CBD-coated pillow cover intervention, the global score (overall sleep quality) decreased from a median of 9.0 (6.0, 12.0) at baseline to 7.0 (6.0, 9.0) after intervention. A lower global score suggests an improvement in sleep quality. The subjective sleep quality score remained consistent in terms of the median at 1.0 (1.0, 2.0) between baseline and after intervention, which indicated improvement in subjective sleep quality, albeit less pronounced. These observed improvements may be partly attributed to the transdermal absorption of CBD, which allows localized and systemic interactions with cannabinoid receptors, potentially influencing relaxation and sleep patterns [32]. Three weeks after using a CBD-coated pillow cover, four participants showed 7.3% improvement in sleep quality. However, in the case of objective sleep quality, there was no significant difference in sleep structure over time, but there was a slight increase in both light sleep and deep sleep duration after using the CBD-coated pillow cover. In the case of mood and lethargy, the total scores for drowsiness and mood (depression and anxiety) were measured over the course of the intervention. CBD’s transdermal absorption through prolonged contact may facilitate localized anti-inflammatory and anxiolytic effects, which could explain the reduction in anxiety and mood improvements observed during the intervention [33]. Compared to improved sleep quality, the CBD intervention significantly reduced anxiety (*p* < 0.005). Reduced anxiety levels may contribute to improved sleep quality, as anxiety often interferes with the ability to fall asleep and maintain restful sleep. A reduction in depressive symptoms could also support better sleep quality, as depression often impacts sleep patterns negatively (e.g., difficulty initiating or maintaining sleep), making the pillow an effective tool for improving overall mental and sleep health.

The participants were classified into two groups based on objective sleep trends: those with an overall improvement in sleep trends and those with unchanged or deteriorated trends. In the comparisons of the basic attributes of these two groups, gender, age, work style and sleeping drug use, smoking and drinking histories did not show significant differences (*p* > 0.05). Only exercise habits showed a significant difference (*p* = 0.03). Specifically, 36.4% of the participants who did not have an exercise habit showed an overall improvement in objective sleep efficiency trends after using a CBD-coated pillow cover, while 63.6% showed either a worsened trend or no change. This suggests that the impacts of the CBD intervention on sleep quality may be less pronounced for individuals with an exercise habit, while those without an exercise habit may experience more significant changes, either positive or negative, in sleep quality.

The CBD-coated pillow cover demonstrated improvements in sleep quality, mood and anxiety, highlighting its potential as a non-invasive intervention for shift workers. Through transdermal absorption, CBD may exert both localized and systemic effects, promoting relaxation and enhancing sleep. Its therapeutic benefits may be particularly valuable for individuals with sleep disturbances associated with stress or anxiety disorders. Additionally, CBD interventions appear to be more effective in individuals who do not engage in regular physical activity, as exercise itself serves as a natural regulator of sleep. However, individual responses to CBD may vary due to differences in metabolism, skin absorption and baseline sleep conditions, resulting in varying degrees of effectiveness. These findings underscore the need for personalized dosing strategies and optimized delivery methods to maximize therapeutic outcomes. Further research is essential to refine CBD-based sleep interventions and tailor treatment approaches to specific patient populations.

### Limitations

This study only tracked and measured the sleep status of individuals using a CBD-coated pillow cover for two weeks; the follow-up effects still need to be tracked continually. In addition, this study did not control for other factors that could affect sleep. It only considered the normal work and rest time of the cases. However, its effects may vary based on individual exercise habits. Further research is needed to explore the potential of CBD in enhancing sleep quality. The small percentage of participants (7.3%) showing significant improvement may suggest that a larger sample size would be needed to detect stronger effects. A longer intervention period might be necessary to see more substantial changes in subjective sleep quality.

## 5. Conclusions

The use of pillow covers coated with CBD enhanced the sleep duration of healthy adults with poor sleep, leading to an increase in duration in the form of deep sleep from baseline to two weeks after intervention. This study also found that the CBD intervention significantly reduced anxiety (*p* < 0.005). Therefore, the findings regarding sleep quality, including light sleep and deep sleep, especially the slight increase in light sleep duration, cannot be extended to other cases of poor sleep quality.

## Figures and Tables

**Table 1:** Demographic characteristics of the subjects (N = 55; missing values: 0).

	N	%
Gender		
Female	45	81.8
Male	10	18.2
Age (years)		
21–30	27	49.1
31–40	20	36.4
≥40	8	14.5
Marital status		
Unmarried	41	74.5
Married	14	25.5
Workplace		
Surgical ward	6	10.9
Internal medicine ward	11	20.0
Acute/intensive care unit	8	14.6
Other	30	54.5
Work style in the past one year		
Fixed day shifts	30	54.5
Fixed evening shift	5	9.1
Fixed night shift	2	3.6
Rotating shift	18	32.7
Work style in the last three months		
Fixed day shifts	32	58.2
Fixed evening shift	7	12.7
Fixed night shift	4	7.3
Rotating shift	12	21.8
Taking adjunct sleeping medications		
No	45	81.8
Yes	10	18.2
Exercise		
No	40	72.8
Yes	15	27.3
Smoking habits		
No	53	96.3
Yes	2	3.6

**Table 2:** The total score at baseline and two weeks after intervention (N = 55; missing values: 0).

Measurements	Baseline	Two Weeks After Intervention	s/t	*p*
**PSQI**				
Global score ^1^	9.0 (6.0, 12.0)	7.0 (6.0, 9.0)	382	<0.001
Subjective sleep quality ^2^	1.0 (1.0, 2.0)	1.0 (1.0, 2.0)	315.5	<0.001
Sleep latency ^3^	3.0 (2.0, 5.0)	2.0 (1.0, 3.0)	315.5	<0.001
Sleep hours (hours)	6.5 (5.5, 6.5)	6.5 (5.5, 6.5)	315.5	<0.001
Sleep efficiency (%)	81.3 (73.7, 91.7)	81.3 (70.3, 87.5)	95.5	0.333
Sleep disturbance ^4^	10.0 (7.0, 13.0)	5.0 (3.0, 10.0)	421.5	<0.001
Sleeping pills were used ^5^	0.0 (0.0, 1.0)	0.0 (0.0, 0.0)	315.5	<0.001
Daytime dysfunction ^6^	2.0 (1.0, 3.0)	1.0 (0.0, 2.0)	314	<0.001
**ESS**	8.18 ± 3.78	5.76 ± 3.80	4.42	<0.001
**HADS**				
Anxiety	10.50 ± 4.43	7.42 ± 4.14	5.68	<0.001
Depression	7.62 ± 3.71	6.38 ± 4.34	3.11	0.003

Due to the skewed distribution of the PSQI global score and its component scores, data are presented as median (first quartile, third quartile), and Wilcoxon signed-rank tests are reported. For the normally distributed ESS and HADS scores, data are presented as mean ± standard deviation, and paired *t*-tests are performed. ^1^. The overall total score represents the total score for PSQI, 0–21 points; the higher the score, the worse the sleep quality; if the score is ≥5 points, sleep quality is classified as poor. ^2^. The subjective sleep quality score ranges from 0 to 3 points: very good (0), fair (1), somewhat poor (2) and very poor (3). ^3^. The total score for sleep latency ranges from 0 to 6 points; the higher the score, the worse the sleep quality. ^4^. The total score for sleep disturbance ranges from 0 to 27 points; the higher the score, the more factors that interfere with sleep, and the worse the sleep quality. ^5^. Sleeping drug use scores range from 0 to 3: never (0), less than once a week (1), once or twice a week (2) and more than three times a week (3). ^6^. The total score for daytime dysfunction ranges from 0 to 6, with higher scores indicating poor sleep quality, which in turn affects daytime life/activities.

**Table 3:** Sleep quality determined using the PSQI at baseline and two weeks after intervention.

	Baseline		Two Weeks After Intervention			
Measurements	n	%	n	%	$x^{2}$	*p*
Sleep quality (PSQI global score)					44.08	<0.001
Good (<5)	0	0.0	4	7.3		
Poor (≥5)	55	100.0	51	92.7		
Subjective sleep quality					10.93	0.091
Very good	1	1.8	4	7.3		
Fair	27	49.1	31	56.4		
Poor	17	30.9	19	34.5		
Very poor	10	18.2	1	1.8		
Sleep latency					15.20	0.019
<15 min	12	21.8	23	41.8		
16–30 min	20	36.4	20	36.4		
31–60 min	15	27.3	10	18.2		
>60 min	8	14.5	2	3.6		
Sleep hours					2.23	0.898
>7 h	7	12.7	5	9.1		
6–6.9 h	27	49.1	24	43.6		
5–5.9 h	18	32.7	22	40		
<4.9 h	3	5.5	4	7.3		
Sleep efficiency					5.79	0.447
>85%	24	43.6	17	30.9		
75–84%	16	29.1	20	36.4		
65–74%	8	14.5	12	21.8		
<65%	7	12.7	6	10.9		
Medication use					10.17	0.118
None	37	67.3	46	83.6		
<1 time/week	9	16.4	4	7.3		
1–2 times/week min	2	3.6	0	0		
>3 times/week	7	12.7	5	9.1		
Daytime dysfunction					12.67	0.049
None	7	12.7	16	29.1		
<1 time/week	27	49.1	32	58.2		
1–2 times/week	16	29.1	7	12.7		
>3 times/week	5	9.1	0	0		

**Table 4:** Sleep hours and sleep distribution at baseline and two weeks after intervention (Fitbit data).

Measurements	Baseline	Two Weeks After Intervention	s/t	*p*
Sleep hours				
TST (min)	362.29 ± 63.72	365.18 ± 63.71	0.60	0.550
WASO (min)	60.1 (46.8, 76.5)	58.3 (46.0, 69.0)	−178.5	0.085
REM (min)	75.22 ± 26.9	75.69 ± 27.2	0.41	0.686
LS (min)	196.21 ± 65.28	206.57 ± 59.15	0.97	0.337
DS (min)	61.97 ± 21.01	64.35 ± 22.19	1.07	0.288
Sleep distribution				
SE (%)	86.3 (83.7, 87.8)	86.8 (85.1, 87.9)	121.5	0.245
WASO (%)	13.7 (12.2, 16.3)	13.2 (12.1, 14.9)	−157.5	0.130
REM (%)	17.33 ± 5.66	17.48 ± 5.14	0.52	0.607
LS (%)	48.1 (42.1, 54.6)	49.3 (45.4, 55.3)	223.5	0.030
DS (%)	15.8 (11.7, 17.9)	15.0 (12.2, 18.2)	−58.5	0.578

Due to the skewed distribution of WASO (min), SE (%), WASO (%), LS (%) and DS (%), data are presented as median (first quartile, third quartile), and Wilcoxon signed-rank tests are reported. For the remaining variables with normal distributions, data are presented as mean ± standard deviation, and paired t-tests are performed. TST: total sleep time. WASO: wakefulness after sleep onset. REM: rapid eye movement sleep. LS: light sleep. DS: deep sleep. SE: sleep efficiency.

## Data Availability

The original contributions presented in this study are included in the article. Further inquiries can be directed to the corresponding author.
